# Can delusions play a protective role?

**DOI:** 10.1007/s11097-017-9555-6

**Published:** 2018-01-25

**Authors:** Rachel Gunn, Lisa Bortolotti

**Affiliations:** 1 Philosophy Department, Edgbaston Campus, University of Birmingham, ERI Building, Edgbaston B15 2TT, UK

**Keywords:** Delusions, Psychosis, Qualitative research, Adaptiveness, Stigma, Mental health, Treatment

## Abstract

After briefly reviewing some of the empirical and philosophical literature suggesting that there may be an adaptive role for delusion formation, we discuss the results of a recent study consisting of in-depth interviews with people experiencing delusions. We analyse three such cases in terms of (1) the circumstances preceding the development of the delusion; (2) the effects of the development of the delusion on the person’s situation; and (3) the potential protective nature of the delusional belief as seen from the first-person perspective. We argue that the development of the delusional belief can play a short-term protective function and we reflect on the implications that this might have for our understanding of psychotic symptoms, for the stigma associated with mental health issues, and for treatment options.

## 1 Introduction

In this paper we provide some support for the view that the development of delusions can count as protective in some circumstances, when viewed from the perspective of the people involved and when related to the life events they previously experienced and those they experienced at the time of developing the delusion. We believe that an investigation into the factors explaining the adoption and maintenance of delusions can contribute to (1) gaining a better understanding of psychotic symptoms, delusions in particular, in the context of detailed first-person accounts; (2) undermining some of the bases for the stigmatisation of people experiencing psychotic symptoms; and (3) informing treatment.

## 2 Delusions as harmful *and* adaptive

Delusions are unusual beliefs that are held with conviction by the person who reports them, but appear implausible to a third party (American Psychiatric Association, DSM-5 2013, page 819). They can be poorly supported by evidence, resistant to counter-evidence, or inconsistent with other beliefs the person has. Clinical delusions can be symptoms of schizophrenia and dementia and might also be experienced by people with other mental health issues as well (such as amnesia and depression).

Clinically significant delusions are harmful: they create anxiety and stress and they impair good functioning in people who report them, contributing to social isolation (McKay et al. 2005, page 315). In the literature it has been suggested that some delusions can have an adaptive function by acting as *defence mechanisms*, protecting people from suffering or from unpleasant truths. Most of the cases discussed in the literature refer to *psychological* adaptiveness rather than *biological* adaptiveness. That is, the claim is that, at the time when they are adopted, some delusional beliefs make the person feel better about a certain situation, temporarily reducing anxiety or stress. Most of the evidence for the psychological adaptiveness of delusions is found in the discussion of so-called “motivated delusions”.

These are delusions, often monothematic, that can be conceived of as a response to physical or psychological trauma, although there are competing hypotheses about their aetiology. Two examples often reported in the philosophical literature are the *Reverse Othello syndrome*, the belief that one’s romantic partner is faithful when she is not, as in Butler (2000), and *anosognosia*, the denial of illness, for instance the denial that one’s limb is paralysed, as in Kortte et al. (2003).

Other delusions, especially those that are systematised and elaborated and can turn into complex, all-encompassing narratives, are not generally regarded as psychologically adaptive. Rather, they are considered as paradigmatic marks of insanity, vividly represented as such in fiction and popular culture. Examples are the delusion of grandeur (the exaggerated belief in one’s self-worth), the delusion of persecution (the belief that one is being threatened and is going to be harmed), and the delusion of reference (the belief that some events are highly significant).

There are few places in the literature where it is suggested that even elaborated delusions can be adaptive. In the prodromal phase of psychosis people experience stimuli that appear to them as exceptionally salient, but do not know how to interpret the stimuli and get anxious (Jaspers 1997, page 98; Kapur 2003). When the delusion is endorsed, some authors describe this as an “a-ha moment” (Conrad 1958), a revelation that puts an end to anxious expectation and makes sense of puzzling experiences (Mishara and Corlett 2009).^1^

Glenn Roberts argues that delusions allow people to attribute meaning to their experiences (Roberts 1992, page 305). Roberts finds that people with elaborated delusions score higher than patients in remission, rehabilitation nurses and Anglican ordinands in the *purpose in life* test and the *life regard* index (Roberts 1991). Both the purpose in life test and the life regard index are widely used and regarded as reliable means for measuring important aspects of meaning and purpose in people’s lives.

The purpose in life test, as the name suggests, measures a person’s experience of meaning and purpose in life (Seeman 1991). It is a 20-item scale and each item is rated on a 7-point scale. Total scores go from 20 (low purpose) to 140 (high purpose). Here are some items: “I am usually: completely bored (1) — exuberant, enthusiastic (7)”; “If I could choose, I would: prefer never to have been born (1) — like nine more lives just like this one (7)”; “As I view the world in relation to my life, the world: completely confuses me (1) — fits meaningfully with my life (7)”.

The life regard index, again as the name suggests, measures a person’s regard for her life (Battista and Almond 1973). It is made of 28 items divided into two subscales: the first measures the ability of the person to see her life within some *framework*, and to have derived a set of life goals or a purpose in life from them; the second measures the degree to which the person sees herself as having fulfilled, or being in the process of fulfilling her life goals. People rate statements based upon their feelings on a 5-point scale ranging from 1 (agree) to 5 (disagree). Sample statements are: “I have a very clear idea of what I’d like to do with my life” and “I don’t really like what I’m doing”.

Roberts concludes that “for some there may be satisfaction in psychosis and that DF [delusion formation] is adaptive” (Roberts 1991, page 19). On Roberts’ view, the delusion can play a defensive function by protecting a person from psychological distress.

Both the specific contents of delusional beliefs and the experience of having found a powerful and comprehensive explanation, accompanied by a conviction of having discovered the truth, could be preferable to confronting reality again. In these circumstances there would be a movement towards elaboration and chronicity. Thus, discrepancies between delusional and real perspectives are likely to be resolved by further elaboration of delusion and adjustment of life circum-stances in order to protect the beliefs from confrontation. A number of theorists with different perspectives have suggested that elaborate delusional systems may, in part, be perpetuated and mediated by the associated psychological benefits. (Roberts 1992, page 305)

Another study focuses on the relationship between delusion formation and “sense of coherence”. The sense of coherence is defined as “a global orientation that expresses the extent to which one has a pervasive, enduring though dynamic, feeling of confidence that (1) the stimuli deriving from one’s internal and external environments are structured, predictable, and explicable; (2) the resources are available to one to meet the demands posed by these stimuli; and (3) these demands are challenges, worthy of investment and engagement.” (Antonovsky 1987, page 91). Moshe Bergstein et al. (2008) find that the sense of coherence is not reduced in people “in acute delusional state” and the sense that one’s life is meaningful might even be enhanced in comparison with the non-clinical population, especially when the delusional system is elaborated. Sense of coherence and meaningfulness are found to correlate with wellbeing. In the transition from the acute state to remission, when conviction in the delusions fades and the new explanation for the delusional experiences involves insight into one’s psychosis, sense of coherence and meaningfulness are reduced, and levels of wellbeing also decrease (Bergstein et al. 2008).

Indeed, in the literature on delusions and hallucinations there are some reports of so-called “successful psychotics” (Hosty 1992, page 373), that is, people who experience positive symptoms of psychosis but do not seem to have their wellbeing significantly compromised by their hallucinations or delusions. In such cases, the delusion seems to confer meaning to people’s experiences (see for instance Jackson and Fulford 1997, pp. 44–45).^2^ Further, we can speculate that cases of “successful psychosis” are much more common than one might think, as people who are not harmed by their delusions due to either good social connections or to the uplifting nature of their delusional experiences are unlikely to seek psychiatric help.

In the literature we briefly reviewed here, there is no attempt to deny the pervasive harmfulness of delusions. But, in different contexts and for different reasons, authors highlight the possibility that delusions might sometimes be adaptive, especially psychologically adaptive. In our paper, we will not present sufficient evidence to either support or challenge the claim that delusions are adaptive. Rather, we argue for the weaker (but related) claim that, from the first-person perspective, delusions can have *a short-term protective function*.

In particular, we argue on the basis of new qualitative research that even elaborated delusions can be conceived of as protective, either because they confer meaning to people’s experiences or because they serve to counteract very unpleasant and traumatic events in a person’s life, sometimes playing the role of reasons to delay or avert suicide attempts. We see them as a sort of *emergency response*. Our suggestion is that delusions, the hallmark of psychosis, may often have some hidden feature that helps explain why they are so resistant to change and why they can become so ingrained in a person’s understanding of the reality surrounding her.

## 3 Methodology

The findings detailed in this paper are part of a larger piece of work aimed at understanding the phenomenology of delusion in general. The purpose of the research was not to investigate whether delusions could be understood in terms of their protective function. However, on analysis, it transpired that a number of the people interviewed had delusions that could be ascribed this function.

A detailed research protocol was written and submitted for National Health Service (NHS) Ethical approval and for NHS Research and Development (R&D) approval by one of us (Rachel Gunn). Research participants were sought via collaboration with local NHS mental health departments. The research was explained to them and they were provided with documents which advised them of the inclusion and exclusion criteria for potential participants.

Inclusion criteria:

- service users within local NHS mental health services
- having active delusion(s)
- able to give informed consent to participate in the study
- able to talk about their experiences
- at minimal risk (of distress) when talking about their experiences
- able to speak English (no translator was provided)
- willing to travel to a suitable NHS location to participate in the interviews.

Exclusion criteria:

- the lead healthcare professional believes he/she would be at risk (of severe distress or suicide) by participating.
- the lead healthcare professional believes that the participant represents a risk to the interviewer.
- the lead healthcare professional believes that the participant is unable to give informed consent to participate in the study.

NHS patients meeting these criteria (as identified by NHS staff) who were interested in participating in the study were introduced to the researcher. Two people discussed the research with the researcher and declined to participate at the time of the initial discussion. Four people discussed the research with the researcher, agreed to participate in principle and then declined to participate for various reasons when the researcher next made contact with them. Four people (one man and three women) agreed to participate and were interviewed. Of the four people interviewed three had what could be described as *protective* delusions. The experiences of these three people are explored in this paper.

The study was qualitative research focusing on what a person’s lived experience was like during the lead-up to, onset of, and current experience of her mental health problems. Each participant was interviewed twice for approximately one hour on each occasion using semi-structured interviews. In the first interview, the participants were asked about their history and the onset of their problems. In the second interview, participants were asked what their experience was like at the time of being interviewed. In practice the interviews were free-flowing with the participant describing their experiences in whatever way came to them. The focus was on what was most important to the participant. The interviews were recorded and transcribed.

Next, for each of the three research participants (who have been called Barbara, Andrew and Alison), we summarise both the significant events preceding the onset of the mental health issue, and the circumstances of the development of the mental health issue. Based on the participants’ first-person accounts, we also speculate as to the ways in which some of their symptoms might be considered protective.

## 4 Barbara

### 4.1 Antecedents to the development of the delusion

One of the research participants, who we have called Barbara, was married to a man who was a serial adulterer. He had already left or threatened to leave Barbara on a number of occasions for other women, she had been desperate for him to stay with her and he had done so each time. When they finally split up Barbara became extremely depressed.

*I was really depressed, really down, really miserable. So I prayed as you do and I asked him to bring my husband back and I said no actually … I’ve asked that time and time again and it’s happened and I said I don’t want it. I said if it’s not good for me I don’t want it. I just want this pain to go away. So… um… I’d already been through a bad time, took an overdose, everything and then all of a sudden… I felt better after I’d prayed.* (Barbara 1, 1:9-15)

Barbara had been through this kind of emotional pain on a number of occasions and while she was feeling this misery she took an overdose (she does not elaborate on this in the interview and it is unclear whether she had taken overdoses before). She did not seek psychiatric help and she prayed for resolution and for the pain to go away.

Barbara suddenly felt better. Her depression and mental anguish disappeared and she was enjoying life. She was engaged with the world, seeing friends, and going out. At the time she was relieved, pleased and happy, but with hindsight she thought this was odd:

…*but I didn’t realise that… the only reason I was feeling… so happy about being single after the… after that massive crash… nobody gets over a breakup in two weeks do they?… not if they really didn’t want it to break up. But… uh… but because God was holding on to me… but I didn’t know that at the time. I just thought… I’m an independent person, I can do this.* (Barbara 2, 19:45-48)

Around this time Barbara started to get the sense that she was being watched and that songs on the radio were presenting important messages to her. She did not seem distressed or worried, perhaps because she was feeling really good and really happy:

… *I’d come home and I’d get the feeling I was being watched… and then the music started talking to me… I don’t know… I can’t explain that very well but I would… I would ask a question and it’d talk to me… It was really weird*. (Barbara 1, 1:28-33)

### 4.2 Development of the delusion

This general feeling that she was being watched and that she was getting messages from the radio persisted. One day she got messages in such a way that made her certain it was supernatural.

… *you don’t imagine that it’s going to be something out of this world. You believe it’s something in this world and you try and explain everything*. (Barbara 1, 1:34-36)

She suddenly realised it must be God. At first she was really happy and she described this realisation (that God was sending her messages) as ‘really lovely.’ This feeling of relief from puzzlement or resolution is a common feature at the onset of the formation of a delusional schema (Mishara 2009; Jaspers 1997). The world, which Barbara experienced as ‘weird’ and found difficult to describe, was suddenly making more sense. An explanation had been found for the sense that she was being watched and for the messages that she had been getting. Then Barbara started getting messages in other ways in the environment, for example, through road signs. Certain words appeared salient and her attention was drawn to them. She found this very difficult to explain. This is a common feature prior to delusion formation (Kapur 2003). It was unclear exactly when it started to happen but at some point she experienced God replying to her questions and prayers. She said that God was talking to her directly ‘by telepathy.’

A few days later Barbara suddenly got messages about a personal decision that she had made a number of years earlier. She made this decision against her better judgement in order to keep her marriage together. Her husband had insisted on it and had threatened to leave if she had not done what he asked. When she received this message Barbara was overwhelmed by fear and guilt and the need to atone.

She thought that giving up smoking and restricting her food intake was important. Eventually she felt she was only ‘allowed’ water, fruit and vegetables. She said at this time she felt like a robot, she was not living, she was just existing.

All this happened over several months and she did not seek help. Eventually she concluded that the Devil must also exist. She could not tell which messages, voices and feeling were God and which were the Devil and she begged God for answers.

*My head was all messed up. I was getting all these messages some from God, some from the Devil, but mostly… it was love and fear, like anger, fear… and guiltiness, when I smoked and ate chocolate as soon as I’d done it I’d feel really really guilty, the guilt would like… eat you up like you’ve done murder or something*. (Barbara 1, 3:32-38)

Barbara experienced intense emotions: love, fear, anger and guilt.^3^ On the one hand, the intense emotions seemed to have something to do with God and the Devil; on the other hand, she seemed to recognise that the emotions did not ‘match’ her initial attempt at reason giving.

Shortly after this things in the environment became ugly and people seemed frightening. She associated this with the Devil. She stopped going out and stopped answering the door to visitors. Eventually Barbara stopped seeing her family, stopped eating and gave all her money away to charity. Her family realised something was seriously wrong.

Barbara was sectioned under the mental health act and detained in hospital against her will. While she was in hospital she drew a paper shield.

*Now, I didn’t make that shield God made it through me and it said all nice things about me, that I was beautiful, I was intelligent, I was important. And it felt really good. I said look what I’ve done look what I’ve done, I was really happy. I was really happy, and… then… as I was writing one verse, I seen* ‘*I am beauty in his sight*’, *I went to write I am beauty in his sight*. (Barbara 1, 6:16-24)

At other times Barbara’s direct communication from God related to positive thoughts about her.

… *as God was talking to me he was making sure that I knew there was nothing wrong with me. And he’s always there, whether I’m right, whether I’m wr… well, he, he says I’m never wrong, God says I’m never wrong*. (Barbara 2, 7:15:18)

*Right, if I say something bad about myself and God will cry and say* ‘*no that’s not right*.’ (Barbara 2, 15:1-2)

*He wouldn’t let me do… he wouldn’t let me do anything that would damage me… something that I wouldn’t be able to live with* (Barbara 2, 16:1-6)

Barbara’s delusion enabled her to feel that she was loved and lovable, that she was intelligent, beautiful, important, and was forgiven for past actions. Her previous depression, guilt, and fear were erased when she realised that God’s love was unconditional. In addition, her tendency to negative self-talk was responded to by God, directly, through telepathy. God told her that the bad things she thinks about herself are mistaken.

In some respects, the content of Barbara’s delusion might be described as protective. At the onset of her delusion she was protected from feelings of being unloved which related to her husband leaving, she was protected from feelings of guilt relating to decisions she made in the past, and she was protected from other negative thoughts she might have about herself. Her delusion might have temporarily prevented a downward spiral of negativity and guilt leading to depression and perhaps suicide. Barbara knew that God and the Devil were involved and that they were both communicating with her in various ways, but she still could not make sense of why she was not allowed to eat or smoke (and others were) and why she felt such intense guilt. At the same time she felt safe knowing that God was with her:

*I know I’m safe wherever I go*. (Barbara 2, 5:32)

… *if it was really upsetting me or really… he wouldn’t let it happen but… like at first when I first got sectioned he lifted me up and talked to me. So that I didn’t feel… I felt really scared at first… but I… I felt scared for a long time, but he was constantly lifting me up in between times*. (Barbara 2, 16:31-35)

Eventually God told her she was his child. She now had an explanation about why God did not want her to eat or smoke - she did not need mortal or human comforts - God would give her everything she needed. She realised that all the other people and the world had been created by the Devil which is why people and places appeared ugly and unpleasant. And she knew that if she could give everything up she would go straight to heaven. This fuller explanation gave her relief from the tension of not knowing what was happening to her, the kind of relief described both in Mishara (2009) and Jaspers ([1963] 1997, page 98).

After a later relapse Barbara described a time of utter bliss. Barbara contrasted her experience of talking to God with the experience of being in the Devil’s world, saying:

*This world wants you to be… It’s the devil’s world and he wants you to be depressed and miserable… He’s… he doesn’t want someone that’s ecstatically happy cos he’s just cruel, he wants everyone to be in pain, upset, annoyed. That’s why this world is as it is*. (Barbara 2, 8:45-52)

There is obviously some truth in her statement. The world is full of suffering and has been particularly difficult (depressing and distressing) for Barbara over the last few years.

Towards the end of the year Barbara started to feel bored which she experienced as painful. The clinical staff looking after her were pleased by this [we can only assume they saw it as evidence of her getting better because her euphoria had diminished]. She went home from hospital and the despair, depression and mental anguish started again. She said:

… *and then the depression, the boredom, the pain. I mean the pain, I was crying all day every day for weeks, months*. (Barbara 2, 8:27-28)

… *the pain was just unbearable, unbearable and again… but this time I didn’t run away from God. I stayed close to God and he said he’ll see me through the pain. Cos I knew that God… if I was feeling that pain then God had to feel that pain*. (Barbara 2, 9:23-27)

Barbara’s euphoria had gone. She was hanging on to the notion that God was with her in order to put up with the anguish and mental pain that she was in. She saw the fact that clinical staff were pleased that she had started to feel bored and distressed as evidence for her schema.

It is completely understandable that Barbara prefers being euphoric and talking to God rather than suffering mental anguish in a world full of Devils. She tried to retain her connection with God, hoping that she could, again, reach the state of bliss that she had been in before she was hospitalised. She concluded that because God is all loving he would not create people to suffer and die. This means that all people (apart from her) must be created by the Devil. For Barbara, staying at home and talking to God is the only activity that has any value. Barbara said that others would be unable to cope with the knowledge of their own mortality:

… *if everyone knew what I knew… then there’d be mass hysteria, everyone would be crying, upset because they know they can’t live, there’s no Heaven for them*. (Barbara 2, 12:14-19)

### 4.3 Protective role of the delusion

It seems that the certainty that she was God’s child and that she was immortal might be protecting Barbara from the mental anguish associated with the inevitability of death. When people were mean to her or when things seemed ugly she could ignore this (she felt it but she did not react to it) because she knew it was the Devil’s work. God helped her with this. Barbara was also protected from feelings of guilt about the past and negative thoughts she might have about herself. Her delusion may have prevented a downward spiral of negativity and guilt leading to depression and perhaps suicide.

It looks like Barbara was now using her schema to help her negotiate all mental distress and perhaps this schema developed and elaborated in order to protect her from the despair associated with insight and recovery (Roberts 1992, p. 305). On the one hand, this might be described as protective as it prevents her from going into a negative downward spiral. On the other hand, now, after a number of years living with this schema it seems that the schema itself is responsible for some of that mental distress.

*I’m telling you. I… I… when it f… when the pain first came along I wished I was normal, the pain was that bad. I wished I could die and be normal… that’s how… I… I wanted to give up my immortality just to die… cos the pain was that bad*. (Barbara 2, 25: 2-4)

*But… the second time I was… no way… no way… I’m happy. I’m God’s child. Even if the pain there I’m God’s child. I’m going to live through this*. (Barbara 2, 25:17-19)

One could argue that the doxastic shear pin *designed* to break when Barbara’s knowledge of the real world threatened to overwhelm her enabled her to experience the world differently and thus protected her, at least in the short term, from unbearable mental distress. The delusion came as a way to spare her the reality of a deeply unpleasant situation. However, since then her way of seeing the world (as a world where she is the child of God and the only one to enjoy immortality) became so entrenched that it might now be seen as the cause of some of her mental distress (because of the sacrifices that she needs to make in order to live up to her role as well as the knowledge that the world is of the Devil’s making).

## 5 Andrew

### 5.1 Antecedents to the development of the delusion

The participant we called Andrew joined a ‘job for life’ workplace at a young age. He was perhaps very naïve about people and the world and says so a number of times while recounting his experiences (e.g., ‘I didn’t know how the world worked’). He immediately found it difficult and unpleasant. He was treated badly and saw others being treated badly. He found it distressing to talk about and avoided doing so, therefore it is unclear exactly what happened (he might have been being bullied at work and perhaps others were too). His father became ill and his mother became his father’s caretaker. He bought a flat in his early twenties and moved out from the parental home to live on his own. Andrew, who was very close to his parents, says he was ‘left alone to my own devices.’ He became fixated on work and concerned about doing well in his job.

Andrew describes his workplace as a ‘hellhole’. He agreed to talk about work but was unable to describe his experience in detail and quickly turned to analogies and generalisations.

*It’s that awful I don’t even like talking about it but I will. It’s that awful. You’ve seen the original* ‘*Planet of the Apes*’… *film, 1964 I think it is with Charlton Heston… and you know how he’s treated during it?*^4^ *Management treat you the… similar to that. That’s how it felt*. (Andrew 1, 6:37-44)

Andrew felt utterly powerless in this situation. He realised that he had no choice about what work he was asked to do, who he worked with, how management treated him and what hierarchy there was. His whole idea of what the workplace should be like and how people should be treated was brought into question.

*So when… managers have more power than the worker they misuse it. It’s human nature. Absolute power corrupts and absolute power corrupts absolutely. If you give people too much power they’ll misuse it*. (Andrew 1, 7:7-10)

*The overall manager of that department was hated, was despised. Now the example she sets, what she does filter down. The other managers copy what she does. And they mis… they mistreat people*. (Andrew 1, 7:16-19)

*Because all you get is disrespect, indecency and it’s not the way that man… it’s not the way to treat people*. (Andrew 1, 8:22-24)

Andrew was intimidated by three managers who were trying to make him take on more work (work that he didn’t think he would be able to do). He became so distressed that he wanted to ‘go nuts’ or ‘smash a chair’ and it took a great deal of effort to stay calm.

The need to do well seemed to become more intense as time went on. Andrew resolved to make things better at work by training in Human Resources (HR) so that he could get out of his immediate environment and improve the workplace environment himself. His ability to look after himself deteriorated as he became more focused on his training course. He realised, with hindsight, that he was not getting enough sleep, was not eating properly and was becoming isolated by prioritising his work and his training course over his social life.

Andrew started behaving oddly. He became obsessed with what others thought and he described this as being paranoid. Although Andrew did not go into detail about what happened at work it seems that his workplace was a distressing and unpleasant place and that it might be necessary to be concerned about what others thought in order to survive. It is also possible that the ‘dangers’ in the workplace were unpredictable, and that navigating the hostile environment required extra vigilance.

Andrew became obsessed with a need to go to the toilet, repeatedly thinking about it and finding that this made it increasingly difficult to concentrate on other things. He also developed checking behaviours. After he has finished and passed his training course this problem escalated. He received a diagnosis of OCD about five months later when he eventually sought help.

For months Andrew was in utter despair. Work was still a ‘hellhole’, he was obsessed with doing well on his training course and doing well at work, he was frightened of the people in the workplace and of the consequences of doing badly or not doing what was asked of him. He was plagued by intrusive thoughts, obsession, and paranoid thoughts.

… *I’ve got brain lock over urine, I can’t move or function without… thinking that I need to go to the toilet literally 24 hours so I needed to take sleeping tablets to get to sleep… it was ridiculous. It was very very frightening*. (Andrew 1, 1:14-21)

… *and then it just got worse into total chaos, um… chaos and torture*. (Andrew 1, 1:38-39)

Andrew became even more socially isolated. Eventually he was unable to work and he would stay at home watching ‘hero’ films. He then had what appeared to be a psychotic episode. He sent an email, called the police, called an ambulance, and ended up as a psychiatric in-patient.

### 5.2 Development of the delusion

Andrew wrote an email about power and injustice saying that he was willing to stand up and challenge various global problems. He sent this to a lot of people. He said that this sense of being directly in touch with God and being his messenger was overwhelming but has now passed. However, as he talked about it, he asserted that he was God’s messenger, wondered if he was mentally ill *and* said this seemed implausible to him because it did not feel ‘medical.’ The feeling of the power that came over him was utterly inexplicable and therefore must be supernatural in some way.

…. *all I know is what happened was is that I was… went on the floor, went on my… this was on my lounge floor, went on the floor and it just felt like evil was trying to turn me into its thing*. (Andrew 2, 5:9-12)

*So it was like a power… it felt like a test from God… that’s what it felt like. Or the devil or whatever you want to call it, but I would say a test from God*. (Andrew 2, 5:38-44)

… *but I was compelled… not compelled… commanded to write. There’s only one way that I can explain it. Imagine someone put your hands on a piano, and… they play it for you. That’s exactly what it felt like. And it felt like God was… on my shoulder or over my shoulder, however you want to coin it, or… inside me*. (Andrew 2, 6:49-53)

*I’m a… I’m a full believer because I know what it felt like. He was with me, over my shoulder or… or yeah, he was with me. It was a force that was so powerful I can’t even explain it to you*. (Andrew 2, 11:47-50)

### 5.3 Protective role of the delusion

One could argue that the *feeling of power* saved Andrew from despair. He had previously felt that his life had lost all meaning as it used to revolve around work and he no longer had a job. His ideas about the way the world should be (just, fair, kind) had been shattered. At this point, he might have felt utterly powerless. To discover that his life had meaning and that his suffering might be ‘for a reason’ might have given him hope for a more positive future. If God had a plan for him then this would resolve the tension he felt about not being able to fully explain, understand, or negotiate the world. It also might have restored a sense of power or agency that had been missing whilst he was suffering in the workplace and suffering due to his OCD.

… *even whilst all the suffering I’d been through, like during it, I’d always thought that this was potentially God’s plan. For me*. (Andrew 2, 11:19-21)

… *you need to take a massive leap of faith. Trust in me. Trust in God and send this e-mail. You are the only person who can do it. You are… a modern day Noah. And what I did is… I sent it to all the most powerful people I know, my mentors who are older, some of these people are HR directors, they’re doctors, world-renowned doctors one of them. And I literally wrote… everything… that it felt I was commanded to by God*. (Andrew 2, 6:30-36)

*I’ve got a huge amount of courage. I’ve always had that even as a child. So if I am… a chosen God’s messenger it’s because of… because of courage predominantly. Cos I do have the courage to do all that is necessary*. (Andrew 2, 13:45-48)

Whilst Andrew had, in some sense, felt that he was not fully in control of typing and sending the email, he also felt that being able to right global wrongs and see that justice was done with God’s help gave his life meaning. This enabled him to restore a sense of agency with regard to things that had previously seemed completely outside his control including how he and others had been treated at work. In this experience, he had become very powerful. He believed that what he had written in the email about certain people actually condemned those people to Hell.

… *that condemns them… at the end of the day. What that shows to me… is that… heaven and hell… they’re off to hell*. (Andrew 2, 18:40-45)

This might have helped restore his previously held ideas about the world being just. If there was no justice or fairness in the workplace, there would be justice through God. Andrew, as God’s messenger, could communicate about the injustice that had been done, condemn those who perpetrated this injustice to Hell, and make sure that justice would be done by ‘doing all that is necessary’.^5^

The sense that his life had meaning and he had suffered for a reason provided relief from perplexity (Mishara 2009; Jaspers [1963] 1997, page 98) as he now understood why he had suffered and why he was overwhelmed by this unusual and inexplicable ‘power.’ His delusion gave him a preferred reality (Roberts 1991) as he now experienced the world as just (as opposed to unjust). The notion that he is God’s messenger and can help to meter out justice enables contact to be maintained with the world whilst incorporating overwhelming and otherwise inexplicable experiences (Mishara and Corlett 2009) as well as, temporarily, restoring power and agency (Bortolotti 2016).

After Andrew’s inexplicable and powerful experience, he recounts his subsequent contact with mental health services in the following way:

*And then I rang the ambulance. They came round and I showed them what I’d sent*. (Andrew 2, 7:35-37)

*So the third test was: are you prepared to… check into a mental institute and you might not come back out again, put your faith in me. And that one was frightening*. (Andrew 2, 8:6-8)

It is not clear whether he went to hospital voluntarily although it seemed that he phoned for the ambulance himself. He has now incorporated being in the psychiatric ward into his delusional schema and describes it as a test from God. He had chosen to undertake this test because he had courage, he was fearless and he was prepared to do ‘all that is necessary’.

## 6 Alison

### 6.1 Antecedents to the development of the delusion

A few years prior to the interview the participant we called Alison experienced problems with a neighbour in her home town (a teenage neighbour regularly set fire to the bins on the street where she lived). Alison later moved away near to where her other family members lived. At this time a family member was falsely accused of sexual assault. Alison attended court every day, became stressed and started to have difficulty sleeping. The family member was found guilty of the crime and subsequently jailed. Alison moved back to where she lived before and started to have problems with her new neighbour who accused her of criminal damage and theft as well as accusing her of the same crime her relative was found guilty of. Her neighbour also expressed her fears that Alison’s relative would come to visit her and sexually assault her children.

Alison had a job in the cash office of a supermarket, there was a problem with her pay and so she left the job. The neighbour told other people who lived on her street that Alison was sacked from the supermarket job because she had been stealing from them.

Alison’s husband was dismissive of her concerns saying that it was ‘all in her head.’ Alison had no one else to talk to. The persecution from her neighbour became unbearable so Alison moved house within the local area to get away from these difficulties (organising the move and the finances alone, with no help from her husband). She then found that her new neighbour was friends with the previous neighbour. On finding this out she took to her bed and stayed there for months, only getting up when she had to (e.g., for hospital appointments or when her children came to visit). She was probably depressed (but this was undiagnosed), she did not seek help and her husband looked after her.

Alison said she had not slept properly for a few years (since her relative was first accused of a crime). She also had atrial fibulation which she found distressing and stressful. This appeared to have had both a physical impact on her and a psychological one. She was feeling panicky when her heart ‘fluttered’ and used breathing techniques to try to ameliorate the ‘flutter’. She was also worried about what the heart condition might mean for her long-term health. She had plaques in a major artery, experienced a severe headache for two weeks, and lost her sight for a few hours – she did not explain this and it is probable she did not fully understand how these things might be related.^6^ When she did tell her cardiologist that she lost her sight for a few hours he admitted her to hospital for observation and tests. She was told that she was likely to require some kind of surgical intervention.

Her husband was also ill and she was worried about him and found this stressful. Other stressors included moving house, having to manage all the household finances and not having anyone to talk to about her concerns and worries.

### 6.2 Development of the delusion

Alison eventually decided that she should go out (she had a hospital appointment and a family event that she wanted to attend). She then had a number of experiences related to hearing the thoughts and conversations of others.

*I got the voices in my head… um… I felt that I could talk to people without moving my mouth, I could hear long distance conversations, but yeah sometimes I still do. And sometimes I walk past people and I feel as though I know what they’re thinking*. (Alison 1, 3:46-49)

*Well, no, that is* true. *I* could *hear people’s thoughts. And I used to go like this as I passed them [puts fingers in ears]. And D said to me one day what on earth are you doing and I said, nothing. They’re having a conversation and I don’t want to hear it*. (Alison 2, 5:4-7)

When asked what she thought when she first ‘heard’ someone’s thoughts, she says:

*That’s when I first thought perhaps D’s right, perhaps I am ill. And then… I just let things go on and things got worse*. (Alison 2, 6:52-52)

On the one hand, at the onset she recognised the bizarre nature of the experience but, as the experience persisted, she was adamant that she was able to read people’s thoughts. This enabled her to keep in touch with a (new) form of reality (Mishara and Corlett 2009; Mishara 2009) which included her ability to read minds and have telepathic conversations. When asked about what happened when she could hear other people’s thoughts and communicate telepathically, she said:

*I think… all around two… three months ago say… it all really started to kick off. And… I don’t know why… because um… there is a drug you can have that can make you do that and whether I thought I’d had that drug, I don’t know*. (Alison 2, 29:34-37)

*I’d just have a conversation with er… like… um… a police officer, you know, cos I thought oh he’s got the same drug [laughs]. So it’s just nonsense in my head*. (Alison 2, 29:47-49)

The police were not getting Alison’s side of the story, but if she could communicate directly with the policeman visiting her neighbour next door using telepathy, then she could be heard and it would be more likely that justice would be done. In this way she might be spared the feelings of powerlessness, the anger over the injustice of the situation, and the fear of the consequences of being falsely accused. The delusion might have enabled Alison to maintain contact with a just world as opposed to a despairing and frightening contact with an unjust world thus enabling her to temporarily regain a sense of agency in a situation where she had felt powerless (Bortolotti 2016).

It is unclear exactly when it started, but at some point Alison developed persecutory delusions (she thought her neighbours were doing things that seem implausible to a third party). Voices and ‘telepathic’ abilities allowed her to ‘hear’ things from her neighbour and social services that were persecutory. On the other hand, her voices and delusional schema about her abilities enabled her to redress the balance by conversing (telepathically) with the police to tell them her side of the story.

### 6.3 Protective role of the delusion

Alison was frustrated that her husband was worried about her, and as she recounts this she says that she had felt suicidal because of the stress relating to the problems with her neighbour.

… *whether he thinks I’m going to commit suicide or whether… he thinks I’m going to walk off or… that’s the last thing in my head cos I don’t feel like that any more… I certainly don’t feel suicidal. I did before, I truly did. In fact I popped 400 pills on the table*. (Alison 2, 3:25-33)

Alison ‘heard’ a conversation through the wall between her neighbour and a policeman which she talked about near the beginning of her first interview.

…*in the end the night I was… the night I was considering to commit suicide she had a phone call from a police officer his name was sergeant J*. (Alison 1, 1:19-21)

… *he said, I can see something happening here, there’s a picture forming, I think you’re trying to frame this lady for something she’s not done. Um… I was laying there listening to this, I’d already popped all these pills*. (Alison 1, 1:25-29)

*Um… and I heard him say to her that um… that they were going to watch it and watch the pattern and see how it formed. She went hysterical at him, she was screaming at him* ‘*arrest her, arrest her, arrest her*’. *They had a big row and he ended up telling her to f off. He slammed the phone down on her and I ended up thinking well perhaps there is someone who believes me. And that stopped me taking the pills and* that *is the truth*. (Alison 1, 1: 33-39)

She reiterated and repeated this point later on in the second interview and went on to say:

*I scooped all those pills up and put them in the bin, and then I got them out the next morning and flushed them down the toilet cos I didn’t want anybody to get hold of them*. (Alison 2, 4:8-10)

There is a real and pragmatic benefit to this ‘voice hearing’ or ‘telepathic’ experience. Alison was enormously relieved that *someone* believed her and this prevented her from taking an overdose of prescription medication. It is almost as if in Alison’s mind the police officer’s intervention, the fact that they defended her against false accusations, was sufficient for her to change her mind about taking the pills.

## 7 Implications of the research

There are at least two competing ways of understanding the conditions in which delusions arise and they are both associated with stigmatisation. In general, we tend to view people with mental health issues as different from people without. One common attitude is to view the mental health issues as part of the person, and to view the person as responsible for the mental health issues (the person is *bad*). The other common attitude is to view the mental health issues as external to the person are and to view the person as transformed and diminished by the mental health issues (the person is *mad*). On the former view, the person’s behaviour is understandable but blameworthy. What is wrong with the person with mental health issues is that she is a *morally defective individual*. On the latter view, the person’s behaviour is not to be blamed but it makes no sense. What is wrong with the person with mental health issues is that she is *damaged machine*.

For instance, the medical model of psychosis suggests that the person is affected by a condition for which she might have a genetic predisposition and for which there are biological markers. Although psychotic symptoms are manifested in behaviour and affect a person’s mental life, the person is no more to blame for developing psychosis than she is for having cancer. Whether this is the best approach to mental health is controversial: should psychosis be understood as a brain disease with an identifiable biological aetiology? Although there is some evidence that there is a genetic link for some conditions such as schizophrenia (e.g., Wicks et al. 2010), clear genetic markers have yet to be identified (Farrell et al. 2015).

The medical model is alleged to reduce stigma as no responsibility is placed on the person for developing psychotic symptoms, but the downside is that stigmatising associations can be made between psychosis and (a) dangerousness, (b) lack of autonomy, and (c) chronicity (Corrigan and Watson 2004; Mehta and Farina 1997). If those who have psychotic symptoms are at the mercy of a biological disease, then (a) they might be unable to control their own behaviour, and therefore be dangerous; (b) they might be lacking in capacity and autonomy, and therefore lose some of their rights and require a third party’s benevolent intervention; and (c) the disease might be seen as chronic and irreversible, making recovery impossible.

A model of psychosis which takes into account not only biological factors, but also social and psychological ones may offer a more balanced account of the person’s capacities and limitations, and may enable us to view and at least partially understand the individual symptom within the context of the person’s overall life experience. The more we know about the experiences of other people, the closer we get to understanding their beliefs and behaviour. Even a very implausible belief can make sense in context: what happened to the person before the belief was adopted? Some strange beliefs with no apparent evidential basis can have grains of truth in them and tell a story.

In the cases we have described in the paper, the delusion is not just a glitch, but its content relates powerfully to significant events in the person’s life and, at the time when it is first adopted, relieves the person of some heavy psychological burden. In particular, in at least some cases, delusion formation can be seen as a short-term protective response to disruptive and traumatising life events. Encouraging this perspective may be a more effective way to break down the stigma associated with psychosis than to describe people with psychosis as damaged machines. Although phenomenological analysis cannot be the sole basis for choosing an approach to delusion formation, it can help us appreciate the context in which delusions are adopted, as it shows us that dire circumstances in the person’s social and physical environment may contribute to the onset of mental health issues. Phenomenological inquiry brings home the fact that anybody may adopt unusual beliefs after experiencing distressing life events, although some individuals may be more vulnerable than others to developing psychotic symptoms.

In the three cases we presented here, the context enables us to see that the delusion is formed following a long period of distress, despair, or depression. Instead of thinking of delusion formation in terms of un-understandable problem with an underlying biological cause which has yet to be found (Jaspers [1963] 1997, page 607) or thinking of psychiatry as synonymous with neuroscience (Tandon et al. 2015; Broome and Bortolotti 2009), we might think of delusion formation as a far more complex phenomenon and of psychiatry as the study of the different factors contributing to that phenomenon as a guide to effective care and treatment options. For instance, we might think that, when people are faced with despair, negative emotions, and suicidal thoughts, the adoption of beliefs that make sense of their experiences can at least temporarily reduce or control the threats they encounter. Whilst our research cannot tell us what the underlying mechanisms are, we can speculate that this process is part of an unconscious defence mechanism, a basic biological response to life-threatening or unbearable distress, or a combination of these.

Such a view has some implications for treatment. If a person’s delusional schema helps her stave off unbearable feelings, and perhaps suicidality, then simply disabusing her of her delusional belief, especially at critical times, might bring about a worse outcome than the presence of psychotic symptoms. When a delusional belief is challenged, something else may need to be put in its place, that is, another response to the person’s crisis that plays the same protective function but is overall less psychologically costly.

In this context, it makes sense to advocate early intervention (prior to the formation of a delusion). This might take the form of some talking therapy helping a person come to terms with her despair or distress. This has wider political or socio-economic implications, because the distress might be related to a person’s lived experience or environment and this might have to change in order for the despair or distress to be ameliorated. But how do we ensure that people facing a psychological crisis seek help before psychotic symptoms emerge? If we want people to seek help earlier, we need to reduce the stigma associated with mental health issues and more data like those analysed in this paper should be gathered and made available in the public domain. If people better understood the nature and the trajectory of mental health issues such as delusions in terms of their context and their potentially protective role, then they might be more able to recognise signs of distress in themselves and others, and become less concerned about the consequences of seeking help.

## 8 Conclusions

In this paper we argued that some psychotic symptoms commonly regarded as marks of madness such as delusions can be construed as protective responses to a psychological crisis, viewed in the context of a person’s life experiences. Thanks to recent empirical work conducted by one of us (Rachel Gunn), we described the development of delusions in three people who experienced adverse circumstances and overwhelmingly negative emotions in their lives, and faced despair or suicidal thoughts.

In Barbara, Andrew, and Alison the development of delusions and hallucinations seems to be a response to a sustained experience of despair and powerlessness. Barbara overcomes unbearable feelings relating to abandonment and guilt and counteracts negative self-talk through her delusional schema. Alison cites what appears to be a delusional or hallucinatory ‘voice hearing’ or ‘telepathic’ experience as being directly responsible for preventing her from taking an overdose of prescription medication at a time when she felt nobody believed her. Andrew regains a sense of agency and meaning from his delusional experience feeling empowered to restore justice in the world. In the short term such experiences can be considered as protective. However, when they persist (as the case of Barbara in particular illustrates), they might add to a person’s psychological distress.

We suggested that an in-depth analysis of first-person accounts of the context in which delusions are formed can help us better understand the nature of psychotic symptoms, undermine some of the bases for the common stigmatisation of people with psychosis, and also inform treatment options.

## Footnotes

1 To claim that delusion formation may be adaptive at the time of adoption does not rule out the fact that most delusions in the long run increase anxiety. For example, paranoid or persecutory delusions, once formed, might induce a person to see previously neutral or benign environmental or social cues as threatening.

2 Our empirical research does not identify people who could be described as “successful psychotics”. All of our research participants have ultimately experienced problems that have caused them to seek psychiatric help or have help sought on their behalf. Here we highlight the notion of the “successful psychotic” simply to show that psychotic symptoms do not always impact negatively on people’s lives.

3 A psychodynamic therapist might think this guilt relates to the past decisions she made against her better judgment in order to stay with her husband and that this is unexamined or unresolved.

4 In the film ‘Planet of the Apes’ human beings are treated like animals, used for slave labour, kept in cages and experiments are done on them.

5 This is a quote from the film ‘Batman Returns’ (Andrew uses this quote a number of times and uses other ‘hero’ film analogies throughout the interviews).

6 This is life threatening. Plaques can break off and cause stroke or heart attack. Alison’s temporary blindness is almost certainly due to a blockage in the retinal vein.
